# The feasibility of atlas‐based automatic segmentation of MRI for H&N radiotherapy planning

**DOI:** 10.1120/jacmp.v17i4.6051

**Published:** 2016-07-08

**Authors:** Kieran Wardman, Robin J.D. Prestwich, Mark J. Gooding, Richard J. Speight

**Affiliations:** ^1^ Department of Medicine University of Leeds Leeds UK; ^2^ Department of Clinical Oncology Leeds Teaching Hospitals Leeds UK; ^3^ Mirada Medical Ltd Oxford UK; ^4^ Department of Medical Physics and Engineering Leeds Teaching Hospitals Leeds UK

**Keywords:** autosegmentation, atlas‐based segmentation, MRI, H&N radiotherapy

## Abstract

Atlas‐based autosegmentation is an established tool for segmenting structures for CT‐planned head and neck radiotherapy. MRI is being increasingly integrated into the planning process. The aim of this study is to assess the feasibility of MRI‐based, atlas‐based autosegmentation for organs at risk (OAR) and lymph node levels, and to compare the segmentation accuracy with CT‐based autosegmentation. Fourteen patients with locally advanced head and neck cancer in a prospective imaging study underwent a T1‐weighted MRI and a PET‐CT (with dedicated contrast‐enhanced CT) in an immobilization mask. Organs at risk (orbits, parotids, brainstem, and spinal cord) and the left level II lymph node region were manually delineated on the CT and MRI separately. A ‘leave one out’ approach was used to automatically segment structures onto the remaining images separately for CT and MRI. Contour comparison was performed using multiple positional metrics: Dice index, mean distance to conformity (MDC), sensitivity index (Se Idx), and inclusion index (Incl Idx). Automatic segmentation using MRI of orbits, parotids, brainstem, and lymph node level was acceptable with a DICE coefficient of 0.73−0.91, MDC 2.0−5.1 mm, Se Idx 0.64−0.93, Incl Idx 0.76−0.93. Segmentation of the spinal cord was poor (Dice coefficient 0.37). The process of automatic segmentation was significantly better on MRI compared to CT for orbits, parotid glands, brainstem, and left lymph node level II by multiple positional metrics; spinal cord segmentation based on MRI was inferior compared with CT. Accurate atlas‐based automatic segmentation of OAR and lymph node levels is feasible using T1‐MRI; segmentation of the spinal cord was found to be poor. Comparison with CT‐based automatic segmentation suggests that the process is equally as, or more accurate, using MRI. These results support further translation of MRI‐based segmentation methodology into clinical practice.

PACS number(s): 87.55.de

## I. INTRODUCTION

Intensity‐modulated radiotherapy (IMRT) for the treatment of head and neck cancers is now established as a standard of care.^(1)^ IMRT provides steep dose gradients to permit dose coverage of target volumes whilst sparing adjacent organs at risk (OAR). The importance of target volume delineation is recognized to avoid the potential for marginal or out‐of‐field treatment failures;^(2)^ additionally, dose sparing of OAR is critically dependent upon accurate OAR delineation.

Data suggest that head and neck cancer target volumes are difficult to contour with higher interobserver variation by comparison with other anatomical sites.^(3)^ Definition of target volumes and OARs is generally a process of time‐consuming manual delineation;^(4)^ in complex head and neck regions complete delineation times of 180 min are reported.^(5)^ This manual process is recognized as being subject to interobserver variability. Although manual delineation is considered the gold standard, there is considerable interest in the use of methods of automatic segmentation to decrease the overall physician‐delineation time and to potentially reduce inherent interobserver and intraobserver variability.^6^, ^7^, ^8^ A role for automatic segmentation is likely to become increasingly important with current interest in adaptive approaches to radiotherapy.^(9)^ Accurate automatic segmentation has been found to be feasible and accurate on CT for OAR with reproducible anatomy/contrast with surrounding tissues.^6^, ^8^, ^10^ A prospective study of atlas‐based automatic segmentation showed an average time saving of 30% for atlas‐based segmentation of OAR followed by a manual edit compared with manual delineation .^(6)^ Similarly, another study showed an approximate time saving of 40% by using automatic segmentation for OAR and lymph node levels.^(8)^ Studies have already demonstrated the role of automatic segmentation for OAR and target volume delineation in adaptive head and neck radiotherapy planning.^(11)^

Currently radiotherapy dose calculation is based upon CT imaging. However, the accuracy of target delineation for head and neck cancers is limited by the lack of soft‐tissue contrast on CT. ^(12)^ CT‐based autosegmentation is limited by the lack of adequate soft‐tissue contrast.^(13)^ By contrast, MRI provides excellent soft‐tissue contrast and there is considerable interest in integrating MRI into the radiotherapy planning process to improve the accuracy of target volume delineation.^(9)^ In addition, functional MRI sequences are promising imaging biomarkers and may additionally be used as a basis for dose painting strategies.^14^, ^15^ MRI is also a superior imaging modality for the delineation of several head and neck OAR.^(16)^ Current research is evaluating the possibility of MRI‐only planning and image‐guided treatment delivery including commercial systems such as the ViewRay MRIdian (ViewRay Inc., Oakwood, Ohio).^17^, ^18^ The direct integration of MRI into the radiotherapy planning process, either by coregistration or as MRI‐only planning, is expected to rapidly increase. A new challenge is the development of automatic segmentation based on MRI with the aim of time saving and also of making use of the soft‐tissue resolution of MRI to improve accuracy of automatic segmentation. Very limited data are available to determine whether automatic segmentation on MRI is feasible, and a comparison of accuracy with established methods of automatic segmentation has not been previously performed.

We have conducted a prospective imaging study evaluating multimodality imaging in radiotherapy planning.^(19)^ The aim of this analysis is to evaluate the feasibility of atlas‐based automatic segmentation of OAR and lymph node levels based on MRI delineation, and to provide a comparison of the accuracy of this process with atlas‐based automatic segmentation using CT images.

## II. MATERIALS AND METHODS

### A. Patients

Fifteen patients entered this single‐center prospective study; one patient withdrew consent prior to imaging. The eligibility criteria for the study were: aged ≥18 years old; WHO performance status of 0−2; histologically proven stage III or IV nonnasopharyngeal locally advanced squamous cell carcinoma of the head and neck region; and a clinical decision to treat with a course of radiotherapy or concurrent chemoradiotherapy with curative intent, and fully informed consent. This study was approved by the Research Ethics Committee (National Research Ethics Committee Yorkshire and the Humber‐Bradford, 11/YH/0212); ISRCTN Registry: ISRCTN34165059.

### B. Image acquisition

#### B.1 FDG PET‐CT

FDG PET‐CT imaging was performed on a 64‐section GE Discovery 690 PET‐CT system (GE Healthcare, Amersham, UK), as previously described.^(19)^ The CT component of the head and neck acquisition was obtained after a 25 s delay following a bolus of 100 mL of iodinated contrast (Niopam 300, Bracco Ltd, High Wycombe, UK) injected at 3 mL/s using the following settings; 120 kV, variable mA (min 10, max 600, noise index 12.2), tube rotation 0.5 s per rotation, pitch 0.969 with a 2.5 mm section reconstruction. The contrast‐enhanced CT component of the PET‐CT scan was acquired with a five‐point thermoplastic radiotherapy immobilization mask fitted and room laser alignment, and was used for radiotherapy planning according to routine clinical protocols.

#### B.2 MRI

Images were acquired on a 1.5 T Siemens Magnetom Avanto (Siemens Healthcare, Erlangen, Germany), as previously described.^(19)^ The patient was positioned in the radiotherapy immobilization device and the axial postcontrast (Dotarem, Guerbet, France) T1‐weighted image (TR=831ms, TE=8.6 ms, 105×2 mm thick contiguous slices, acquired voxel size=0.9×0.9×2.0 mm) was performed.

### C. Manual delineation

Manual delineation was performed by a medical student and checked by a senior clinical oncologist on the MRI T1 postcontrast sequence and on the contrast‐enhanced CT images separately. OAR delineated were left and right parotid glands, left and right orbits, brainstem, and spinal cord in all patients. Brainstem was delineated from the junction of pons and medulla down to the foramen magnum. Spinal cord was delineated from below foramen magnum down to the inferior aspect of the C6 vertebral body. As a proof of principle the left nodal level II was delineated in all patients with no radiological evidence of lymph node in the left neck according to the 2013 DAHANCA, EORTC, HKNPCSG, NCIC CTG, NCRI, RTOG, TROG consensus guidelines.^(20)^

### D. Atlas definition and automatic segmentation

Contours were generated for OAR (left and right parotid glands, left and right orbits, brainstem, spinal cord) in all patients and for the left lymph node level II in patients with no radiological evidence of lymph node disease in the left neck using a ‘leave‐one‐out’ multiatlas method of automatic segmentation. For each patient the leave‐one‐out approach selected each patient in turn as a target patient and used all other patients in the study as atlas patients. A deformable image registration was then performed between each atlas patient to the target patient. Registrations were performed using commercial image registration software (Mirada RTx v1.6, Mirada Medical, Oxford, UK), using standard registration presets intended for same‐patient registrations. Once registered, structures were transposed from each atlas patient to the target patient using the deformation vector field. A form of majority voting was used to combine structures from each atlas to leave one automated structure. This was performed on MRI and CT separately.

### E. Comparison metrics

Accuracy of the automatic contours was determined by comparing them to the manual contours, regarded as the gold standard in this study. This is because delineation of OAR remains the ‘gold standard’ in terms of accuracy.^(21)^ Agreement of autocontours with manually delineated contours was assessed using positional contour comparison metrics calculated by ImSimQA v3.1.5 (OSL, Shrewsbury, UK): mean distance to conformity (MDC); DICE index; sensitivity index (Se Idx); and inclusion index (Incl Idx). MDC is the mean of distances to agreement between two contours and is defined as the mean distance that each point on the test contour would have to move to overlap with the nearest point on the reference contour; as agreement improves, MDC decreases.^(22)^ DICE index, Se Idx, and Incl Idx are all measures of overlap. The DICE index produces output values between 0 and 1, where 0 represents two contours with no overlap and 1 represents two contours that are perfectly overlapping; it is defined in Eq. (1) as twice the overlap volume between two structures divided by the addition of the two individual volumes, $V_{\text{test}}$ is the volume of the test structure and $V_{\text{reference}}$ is the volume of the reference structure:^(23)^

(1) $$DICE=2\frac{V_{test}\cap V_{reference}}{V_{test}+V_{reference}}$$

The Se Idx and Incl Idx calculate the overlap volume between the test and reference contours as a percentage of either the test or reference contours for Incl Idx and Se Idx, respectively (see Eqs. (2) and (3)). Both indices vary between 0 and 1, with a value of 1 indicating a perfect overlap. The *Se Idx* is a measure of the probability that the automatic contour matches the reference manual contour. *The Incl Idx* is the probability that a voxel of the autocontour is really a voxel of the reference manual contour.

(2) $$Se.Idx=\frac{V_{test}\cap V_{reference}}{V_{reference}}$$

(3) $$Incl.Idx=\frac{V_{test}\cap V_{reference}}{V_{test}}$$

### F. Statistical methods

A two‐tailed paired *t*‐test was used for significance testing between volume and positional metrics. Results were considered significantly different if the p‐value was <0.05.

## III. RESULTS

**Figure 1:**
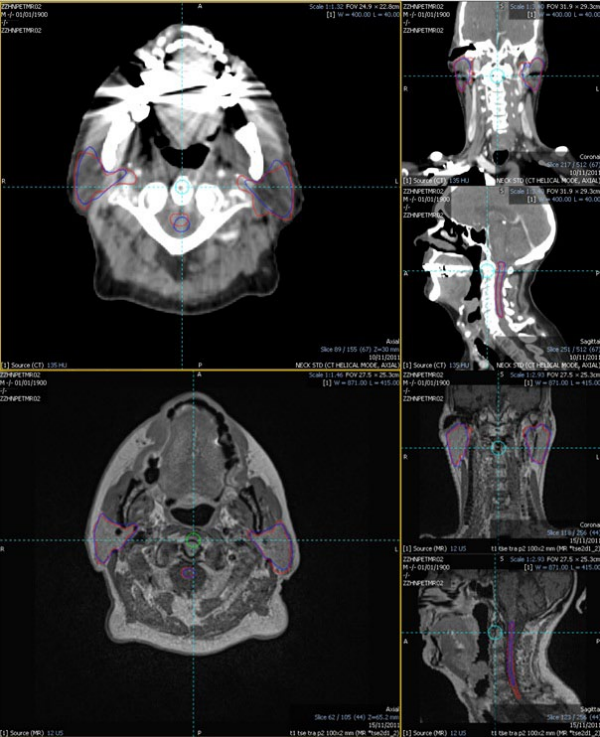
Example manual contours (red) and autocontours (blue) for the spinal cord as well as left and right parotids for Patient ‣2. Top images are CT showing large dental artifacts and poor autocontours, and bottom images are MRI showing more accurate autocontours.

Fourteen patients with nonnasopharyngeal squamous cell carcinoma underwent pretreatment MRI and PET‐CT imaging in an immobilization mask. Median age was 56 yr (range 39–69). Eleven patients had oropharyngeal carcinoma and three patients had laryngohypopharyngeal carcinoma. One had stage III and 13 patients had IV disease . Nine patients had no radiological evidence of nodal disease in the left neck; scans from these nine patients were used for contouring the left level II nodal structure. Figure 1 illustrates example manual and autocontours generated on the CT and MRI scans.

### A. Volume analysis

Mean volumes of each OAR and the left level II structures both manually and automatically delineated on MRI and CT are shown in Table 1. On MRI, the right orbit was significantly larger for the automatically segmented structures compared with the manually segmented ones, whilst the brainstem, spinal cord, and left lymph node level II were significantly smaller. For CT based delineation, the right orbit was significantly larger when automatically segmented, and the brainstem and left parotid gland were significantly smaller.

**Table 1:** Mean volume of both manual contours and autocontours. Result were considered significantly different if the p‐value was <0.05.

	*Manual Contour Volumes* (${\text{cm}}^{3}$)			*Autocontour Volumes* (${\text{cm}}^{3}$)				
*Structure*	*CT*	*MRI*	*p‐value between CT and MRI Manual Contours*	*CT*	*MRI*	*p‐value between CT and MRI Autocontours*	*p‐value between Manual and Autocontours on CT*	*p‐value between Manual and Autocontours on MRI*
LO	4.78	6.02	<0.001	5.2	6.3	<0.01	0.05	0.06
RO	4.78	5.87	<0.001	5.16	6.26	<0.01	0.034	0.03
LPG	25.6	26.55	0.39	21.51	29.08	<0.01	0.02	0.21
RPG	25.86	27.99	0.08	22.44	29.71	<0.01	0.07	0.24
BS	2.94	3.55	0.01	2.72	2.51	0.24	0.04	<0.01
SC	6.02	5.66	0.24	5.98	1.52	<0.01	0.60	<0.01
L II	30.18	30.89	0.50	27.59	24.55	0.08	0.13	0.04

LO=–left orbit; RO=right orbit; LPG=left parotid gland; RPG=right parotid gland; BS=brainstem; SC=spinal cord; L II=left lymph node level II.

Significant differences were found in the volume of manually delineated orbits and brainstem on MRI compared with CT. Automatically segmented volumes for the orbits and parotids were significantly larger for MRI compared with CT, whilst the spinal cord was significantly smaller.

### B. Positional analysis

The mean positional contour comparison metrics are shown in Table 2. The process of automatic segmentation was significantly more accurate on MRI‐compared CT (using manually delineated structures on MRI and CT as a ‘gold standard’, respectively) for orbits, parotid glands, and left lymph node level II by multiple positional metrics. There was a trend for automatic segmentation of the brainstem on MRI to be more accurate, although differences in positional metrics were only significant for the Incl Idx. By contrast, spinal cord was significantly more accurately autosegmented on CT compared with MRI; by MDC, DICE and Se Idx autosegmentation of the spinal cord on MRI was very inaccurate.

**Table 2:** Mean CT and MRI results comparing the autocontours to the manual contours for MDC, DICE, Se Idx, and Incl Idx. Result were considered significantly different if the p‐value was <0.05.

	*MDC (mm)*			*DICE*			*Se Idx*			*Incl Idx*		
	*CT*	*MRI*	*p‐value between CT and MRI*	*CT*	*MRI*	*p‐value between CT and MRI*	*CT*	*MRI*	*p‐value between CT and MRI*	*CT*	*MRI*	*p‐value between CT and MRI*
LO	3.45	2.04	<0.01	0.87	0.91	<0.01	0.91	0.93	0.04	0.83	0.89	<0.01
RO	3.33	2.13	<0.01	0.87	0.9	<0.01	0.91	0.93	0.07	0.84	0.87	0.08
LPG	6.66	4.79	<0.01	0.76	0.79	0.12	0.71	0.84	<0.01	0.83	0.76	<0.01
RPG	6.23	5.15	<0.01	0.75	0.79	<0.01	0.71	0.82	<0.01	0.82	0.77	0.05
BS	4.26	3.19	0.02	0.69	0.73	0.49	0.69	0.64	0.34	0.74	0.89	0.01
SC	3.51	17.5	0.01	0.8	0.37	<0.01	0.8	0.26	<0.01	0.81	0.93	<0.01
L II	5.57	3.95	0.45	0.78	0.8	0.01	0.81	0.76	<0.01	0.76	0.84	0.69

LO=–left orbit; RO=right orbit; LPG=left parotid gland; RPG=right parotid gland; BS=brainstem; SC=spinal cord; L II=left lymph node level II.

## IV. DISCUSSION

Atlas‐based segmentation is a method by which a reference or atlas image is registered to a new image and the corresponding structures transposed on the new image. The quality of structure segmentation is dependent upon multiple factors, including the quality of the atlas, the similarity of the structure in the atlas and the new image, and the contrast within the images by which the structure can be defined.^(13)^ The use of multiple atlas images aims to overcome anatomical variations between different patients, using a voting methodology by which individual voxels are placed within or outside the segmented structure.^13^, ^24^ Atlas‐based segmentation using a CT atlas has now entered routine clinical practice for segmentation of OAR and lymph node target volumes on planning CT scans.^6^, ^8^, ^13^ For head and neck radiotherapy delineation, these techniques have demonstrated time sparing benefits^6^, ^8^ and a reduction in interobserver variability.^(25)^ It remains clear from these studies that automatic segmentation is not sufficiently accurate to remove the need for a manual editing process.^6^, ^8^, ^26^

There is considerable enthusiasm to increase the role of MRI in head and neck radiotherapy planning, making use of superior soft‐tissue contrast to improve target volume and OAR delineation; in addition, functional MRI sequences may allow individualization/adaptation of treatment approaches.^9^, ^14^ The use of MRI in radiotherapy planning is expected to continue to increase, either using coregistration software or, in the future, as MRI‐only planning. Therefore, it is important to determine whether methods of automatic segmentation which have been successful with CT‐based planning can be translated to MRI. Only limited data are available assessing the feasibility of accurate MRI‐based automatic segmentation.^(27)^ A previous study has shown that a multiatlas‐based registration combined with machine learning‐based segmentation can be used to accurately segment submandibular glands, parotid glands, and bone;^(28)^ the same group found in a study of three patients that level II lymph nodes could be automatically segmented with reasonable accuracy on on‐treatment MRI scans.^(28)^ It can additionally be hypothesized that the improved soft‐tissue contrast provided by MRI may serve to improve the accuracy of segmentation of both OAR and target volumes; this has not been tested prior to this study.

Our data have demonstrated that atlas‐based automatic segmentation based on MRI is able to accurately segment multiple OAR (orbits, parotids, brainstem) and a lymph node level, based on multiple metrics of volume and positional analysis. For example, the DICE index for these structures was high, ranging between 0.73−0.91. By contrast, the data clearly show that the methodology failed to accurately segment the spinal cord. A potential reason for this may be that the spinal cord is a long structure and registration on MRI may not be as accurate over such a length. An alternative explanation may be to do with image registration algorithms having a capture range;^(29)^ a long structure, such as the spinal cord, may be beyond this capture range, meaning any registration algorithm is unlikely to correctly account for any deformation over the whole volume.^(29)^ The process used to generate autocontours was based on a preset intended for intrapatient registrations, not interpatient as used in the current work. It is expected that the algorithm could be optimized (by altering the regularization and capture range) to improve results, however this is not possible in the commercial software package we were using and, therefore, was beyond the scope of the undertaken work.

For comparative purposes, we have performed a similar atlas‐based automatic segmentation on the same structures delineated on CT. This method is comparative and the ‘gold standard’ for CT and MRI are separately defined, in the absence of an absolute ground truth that can be derived from CT or MRI. However, this method is instructive to compare the accuracy of the automatic segmentation process on MRI with a method that is already in clinical use for CT‐based radiotherapy planning. It is interesting to note that, on multiple positional parameters for structures other than the spinal cord, the process appears similar or more accurate when performed MRI. This not only validates the feasibility of MRI‐based, atlas‐based automatic segmentation, but additionally provides encouragement that the process has the potential to harness the soft‐tissue contrast of MRI to come closer to the ground truth.

There are several limitations to this study. There is no consensus on the appropriate size or inclusion criteria of atlas for atlas‐based segmentation.^(13)^ For the analysis of the left level II lymph node volume, only nine patients were suitable after the exclusion of patients with nodal disease in the left side of the neck. The results of the positional analysis, however, suggest that the use of a smaller atlas was not detrimental. The assessment of the accuracy of autosegmentation is a challenging task; it is recognized that methods of overlap assessment and distance to agreement do not necessarily result in similar conclusions.^(13)^ In the absence of a standard methodology for this assessment, we have utilized multiple positional parametrics. A multitude of anatomical and functional MRI sequences are now available, several of which are of interest to improving delineation or for guiding adaptive strategies based upon functional characteristics. This study was based only on T1‐weighted sequences; these data may not be extrapolatable to alternative sequences which may also have a role in radiotherapy planning. An additional limitation is that we did not include an assessment of potential time saving of MRI‐based autosegmentation. By contrast with CT‐planning, our experience of MRI delineation is inevitably limited; therefore, we did not feel that assessment of the time of delineation in these circumstances would be meaningful.

## V. CONCLUSIONS

These data demonstrate the feasibility of accurate atlas‐based automatic segmentation of OAR and lymph node levels using T1‐weighted MRI; segmentation of the spinal cord was found to be inaccurate. Comparison with CT‐based automatic segmentation suggests that the process is equally or more accurate using MRI. These results support further translation of MRI‐based segmentation methodology into clinical practice.

## COPYRIGHT

This work is licensed under a Creative Commons Attribution 3.0 Unported License.

## Supporting information

Supplementary Material
