# Supplementary material for: The feasibility of atlas‐based automatic segmentation of MRI for H&N radiotherapy planning
Source: J Appl Clin Med Phys. 2016 Jul 8;17(4):146–54. doi: 10.1120/jacmp.v17i4.6051 (PMC5690045; doi:10.1120/jacmp.v17i4.6051)
Supplement: Supplementary file 1 — Supplementary Material [file ACM2-17-146-s001.doc]

**The feasibility of atlas-based automatic segmentation of MRI for H&N radiotherapy planning**

**Kieran Wardman**

*Department of Medicine, University of Leeds, UK*

**Robin JD Prestwich**

*Department of Clinical Oncology, Leeds Teaching Hospitals, UK*

**Mark J Gooding**

*Mirada Medical Ltd, Oxford, UK*

**Richard J Speight**

*Department of Medical Physics and Engineering, Leeds Teaching Hospitals, UK*

[richard.speight@nhs.net](mailto:richard.speight@nhs.net) (corresponding author)

Running title: MRI autosegmentation for H&N
